# The Sensitivity of Grating-Based SPR Sensors with Wavelength Interrogation

**DOI:** 10.3390/s19020405

**Published:** 2019-01-19

**Authors:** Jianjun Cao, Yuan Sun, Yan Kong, Weiying Qian

**Affiliations:** School of Science, Jiangnan University, Wuxi 214122, China; jndxsy1225@126.com (Y.S.); ykong@jiangnan.edu.cn (Y.K.); wyqian@jiangnan.edu.cn (W.Q.)

**Keywords:** surface plasmon resonance, metal grating, sensitivity enhancement, biosensor

## Abstract

In this paper, we derive the analytical expression for the sensitivity of grating-based surface plasmon resonance (SPR) sensors working in wavelength interrogation. The theoretical analysis shows that the sensitivity increases with increasing wavelength and is saturated beyond a certain wavelength for Au and Ag gratings, while it is almost constant for Al gratings in the wavelength range of 500 to 1000 nm. More importantly, the grating period (*P*) and the diffraction order (*m*) dominate the value of sensitivity. Higher sensitivity is possible for SPR sensors with a larger grating period and lower diffraction order. At long wavelengths, a simple expression of *P*/|*m*| can be used to estimate the sensor sensitivity. Moreover, we perform experimental measurements of the sensitivity of an SPR sensor based on an Al grating to confirm the theoretical calculations.

## 1. Introduction

Biosensors based on surface plasmon resonance (SPR) have received much attention since their first application in gas sensing in 1983 [1]. The ability to detect very small changes in the refractive index makes SPR sensors a good platform to characterize gases, chemical molecules, and living cells, showing advantages of label-free and real-time detection [2,3,4,5]. Because the surface plasmon wave vector is larger than the free space one, excitation of SPR needs the help of high index optical elements like a prism [6,7], patterned nanostructures like diffraction grating [8,9], or optical fibers [10,11,12,13,14,15]. Nowadays, the prism-based SPR sensors are developed into commercial products due to their extremely high sensitivity. However, the use of prisms makes this design inevitably bulky. In recent years, there is growing interest in developing integrated SPR sensors to meet the requirements of portable point of care applications. Grating-based SPR sensors are suitable candidates for compact or integrated biosensors [16,17,18,19,20,21]. For example, an integrated chip with a gold grating substrate, which is only 1 cm^2^ in its size, is successfully fabricated [18]. However, the sensitivity of this type of SPR sensors has not been fully explored.

The sensitivity (*S*) of a sensor is referred to by the ratio between the shift of the wavelength or angle and the shift of the refractive index. Theoretical analysis of the sensitivity is important for optimal design of sensors [22,23,24,25,26,27,28]. Previous literature has reported the dependence of sensitivity on the system parameters in different sensing methods. For prism-based SPR sensors in wavelength mode, *S* increases as wavelength increases, and silver films have higher sensitivity than gold films [29,30]; for that in angular mode, *S* is high at short wavelengths and tends to be constant at long wavelengths. For grating-based SPR sensors in angular mode, in the wavelength range of 600–1000 nm, *S* increases as wavelength increases and grating period decreases for the first diffraction order; for that in the wavelength mode, which is very close to the condition discussed in this paper, Homola et al. find that *S* approaches $\lambda /n_{a}$ at long wavelengths, where λ is the wavelength and $n_{a}$ is the refractive index of the analyte [30]. However, in their theoretical derivation the incident angle in the analyte was treated as a fixed value, which was hard to be realized in practical experiments. In practical experiments, the incident angle in air is fixed, while the angle in the analyte varies with its refractive index according to Snell’s law.

In this paper, we derive the theoretical expression for the sensitivity of grating-based SPR sensors working in wavelength interrogation. The influences of system parameters, namely the operating wavelength, the grating period, the type of metal, and the refractive index of the analyte, on the sensitivity are comprehensively discussed. Furthermore, we perform experimental measurements of the sensitivity of a SPR sensor based on an Al grating to confirm our theoretical calculations.

## 2. Theoretical Analysis

**Figure 1:**
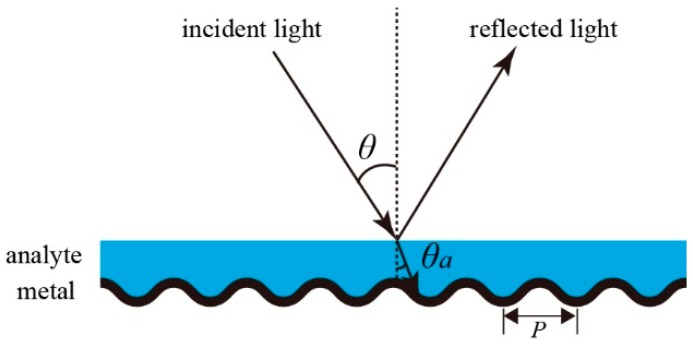
Schematic diagram of the grating-based surface plasmon resonance (SPR) sensor.

The considered optical geometry of the SPR sensor is sketched in Figure 1. It consists of a metal grating and a fluidic channel. The metal grating has a sinusoidal profile with a period of *P*. The analyte filling in the fluidic channel has a flat top surface. To excite SPR at a given wavelength, a collimated white light with TM polarization (electric vector perpendicular to the grooves of the grating) is the incident on the sensor at a specific angle of θ. The reflected light is then sent to a spectrometer that measures the reflection spectra. The excitation of SPR will be observed as an absorption dip in the spectra.

The key to excite SPR is wave vector matching of the incident light and the surface plasmon polariton. For a surface plasmon propagating at the interface between a metal layer with dielectric constant of $\varepsilon_{m}$ and a dielectric layer with dielectric constant of $\varepsilon_{d}$, its wave vector can be expressed as:

(1) $$k_{sp}=\frac{2\pi }{\lambda }\sqrt{\frac{\varepsilon_{m}\varepsilon_{d}}{\varepsilon_{m}+\varepsilon_{d}}}.$$

This wave vector is always greater than the in-plane wave vector of the incident light, which is $k_{x}=\frac{2\pi }{\lambda }n_{a}\sin\theta_{a}$ ($n_{a}$ is the refractive index of the analyte and $\theta_{a}$ is the angle in the analyte). In grating coupling scheme, the wave vector of the diffracted beam ($k_{d}$) is the sum of $k_{x}$ and the grating wave vector [3,30]:

(2) $$k_{d}=\frac{2\pi }{\lambda }n_{a}\sin\theta_{a}+m\frac{2\pi }{P},$$

where m=0, ±1, ±2, … is the diffraction order. Therefore, the SPR excitation condition is now $k_{d}=k_{sp}$, which can be written as:

(3) $$n_{a}\sin\theta_{a}+m\frac{\lambda }{P}=\pm \sqrt{\frac{\varepsilon_{m}n_{a}^{2}}{\varepsilon_{m}+n_{a}^{2}}}.$$

In this equation, $\varepsilon_{d}$ is replaced by $n_{a}^{2}$, the sign ‘±’ on the right side corresponds to surface plasmons propagating along the positive or negative direction. In practical experiments, the incident angle in air (θ in Figure 1) is fixed, while $\theta_{a}$ varies with $n_{a}$ according to Snell’s law, which is $\sin\theta =n_{a}\sin\theta_{a}$. Thus, Equation (3) should be modified as:

(4) $$\sin\theta +m\frac{\lambda }{P}=\pm \sqrt{\frac{\varepsilon_{m}n_{a}^{2}}{\varepsilon_{m}+n_{a}^{2}}}.$$

**Figure 2:**
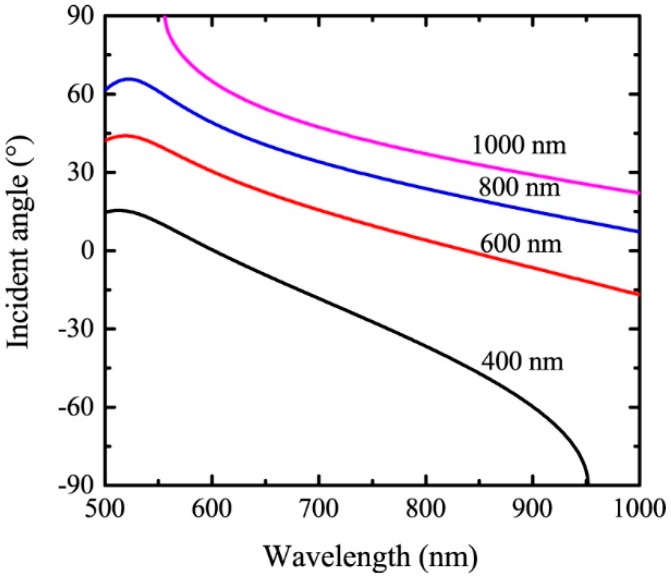
The angle of incidence as a function of the resonant wavelength for four different grating periods. The material of the grating is Au and $n_{a}=1.34$, m=1.

Here, we take particular concerns in the spectra range of 500 to 1000 nm, where high-performance silicon detectors are applicable. Assuming that the material of the metal grating is Au and the refractive index of the analyte is 1.34, the incident angle to excite SPR is calculated as a function of the resonant wavelength for four different grating periods of 400 nm, 600 nm, 800 nm, and 1000 nm for the first diffraction order. The results are shown in Figure 2. Generally, the angle decreases with increasing wavelength. Due to the constraint of |sinθ|≤1, SPR cannot be excited at long wavelengths for *P* = 400 nm, as well as short wavelengths for *P* = 1000 nm.

By differentiating Equation (4) in λ and $n_{a}$, the analytical expression for the sensor sensitivity is obtained as:

(5) $$S=\frac{d\lambda }{dn_{a}}=\frac{{\left(\frac{\varepsilon_{m}}{\varepsilon_{m}+n_{a}^{2}}\right)}^{\frac{3}{2}}}{\frac{\left|m\right|}{P}-\frac{n_{a}^{3}}{2\sqrt{\varepsilon_{m}}{\left(\varepsilon_{m}+n_{a}^{2}\right)}^{\frac{3}{2}}}\;\frac{d\varepsilon_{m}}{d\lambda }}.$$

For simplicity, we use the Drude model for the dielectric constant of metal to calculate $\frac{d\varepsilon_{m}}{d\lambda }$. The Drude model is

(6) $$\varepsilon_{m}\left(\omega \right)=1-\frac{\Omega_{P}^{2}}{\omega \left(\omega -i\Gamma_{0}\right)},$$

where ω=2πc/λ, $\Omega_{P}=\sqrt{f_{0}}\omega_{P}$ is the plasma frequency, $f_{0}$ is the oscillator strength, and $\Gamma_{0}$ is the damping constant. The values of $f_{0}$, $\omega_{P}$, and $\Gamma_{0}$ are obtained from [31,32]. Differentiating Equation (6) in λ, we obtain

(7) $$\frac{d\varepsilon_{m}}{d\lambda }=\frac{\Omega_{P}^{2}\omega^{2}\left(i\Gamma_{0}-2\omega \right)}{2\pi c{\left(\omega^{2}-i\Gamma_{0}\omega \right)}^{2}}.$$

**Figure 3:**
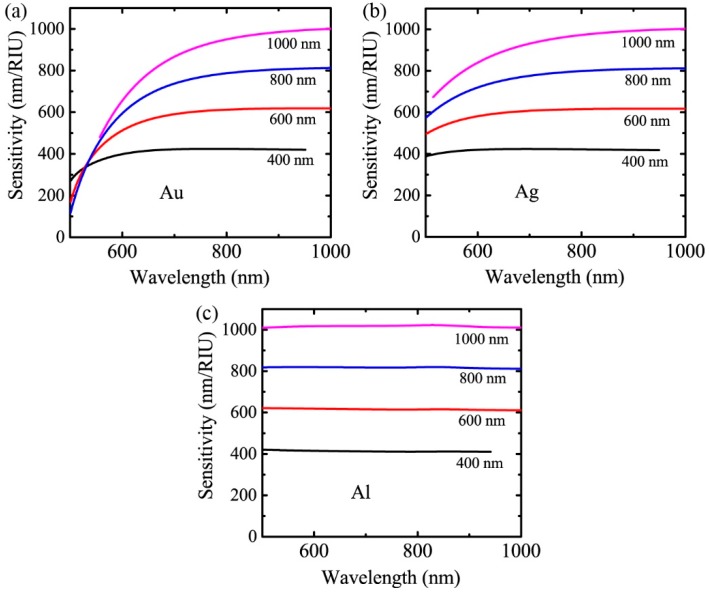
The theoretical sensitivity of the grating-based sensor as a function of the wavelength for four different periods with $n_{a}=1.34$, m=1. The materials for the gratings are (**a**) Au, (**b**) Ag, and (**c**) Al, respectively.

By substituting $\frac{d\varepsilon_{m}}{d\lambda }$ using Equation (7) and $\varepsilon_{m}$ using Lorentz-Drude model into Equation (5), the value of the sensitivity can be calculated. Figure 3 shows the calculated sensor sensitivity versus the wavelength for grating periods of 400 nm, 600 nm, 800 nm, and 1000 nm with system parameters of $n_{a}=1.34$, m=1. The materials for the gratings in Figure 3a–c are Au, Ag, and Al, respectively.

From Equation (5), we can find that |m|/P dominates the behavior of the sensor sensitivity for a given material. At long wavelengths, the numerator in Equation (5) approaches 1 and the second term in the denominator is much smaller than the first term. Therefore, the sensitivity at long wavelengths can be estimated by P/|m|. This is very different from the prediction of $\lambda /n_{a}$ calculated in [30]. According to [30], the sensitivity increases with wavelength and is weakly dependent on the diffraction order and period. This leads to the optimization strategy of designing sensors working at the longest possible wavelength and ignoring the choice of grating period and diffraction order. However, for the optical geometry in our case, *S* increases as the grating period increases and the diffraction order decreases. The wavelength dependence of *S* varies with the material of the grating. *S* increases with wavelength and is saturated beyond a certain wavelength for Au and Ag, while it does not change considerably with wavelength for Al. Thus, the sensors should not only be designed to work at longer wavelengths but also have larger grating periods and work at the ±1st diffraction order.

The large difference between the sensitivity calculated in this paper and in [30] originates from the different models that are considered. In our paper, the light source is outside the analyte channel, while the light source should be inside the analyte channel in [30]. The SPR excitation condition in our case is Equation (4) and that in [30] is Equation (3). Therefore, the expression of the sensitivity derived here is different with that in [30].

Our model has its limits. The incident angle cannot exceed 90 degrees, so Equation (4) should satisfy the constraint of |sinθ|≤1. This constrain limits the grating period that can be applied. For example, at λ=800 nm and $n_{a}=1.34$, the period of Al grating should be in the range of 340 nm to 2267 nm.

**Figure 4:**
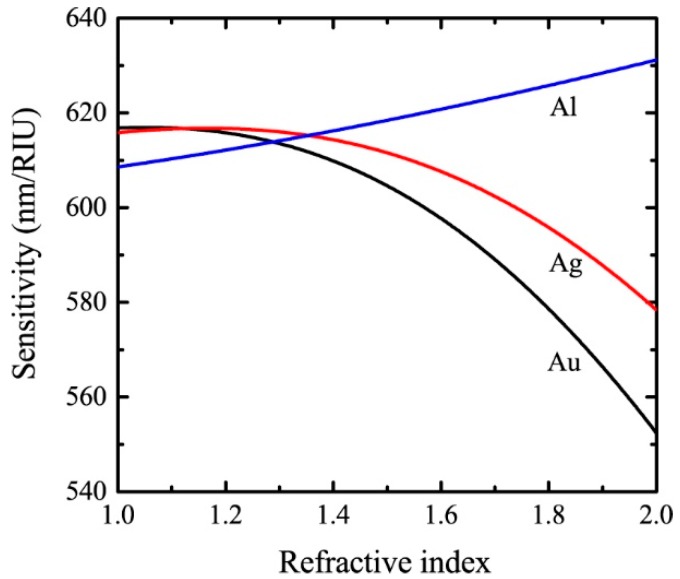
The refractive index dependence of the sensitivity for SPR sensors with a period of 600 nm and a resonant wavelength of 800 nm.

The dependence of the sensitivity on the refractive index of the analyte in the range of 1 to 2 is depicted in Figure 4. In the calculations, the grating period and the resonant wavelength are assumed to be 600 nm and 800 nm, respectively. As can be seen, *S* decreases with increasing $n_{a}$ for Au and Ag, while increases with increasing $n_{a}$ for Al. Since the real parts of the dielectric constants of these metals are negative, both the numerator and the denominator in Equation (5) increase with the refractive index of the sensing medium. The dependence of sensitivity on the refractive index is a result of the competition between the increasing speeds of the numerator and the denominator.

Our theoretical calculations can be compared with the experimental results in previous literatures. For an Au grating with a period of 320 nm, sensitivities of 425 nm/RIU and 360 nm/RIU were obtained for 100 nm and 50 nm film thickness, respectively [18]. With the same grating period, an Ag grating-based sensor exhibited sensitivity of 356 nm/RIU [17]. Our theoretical analysis is also valid for sensors that measure the enhanced transmission spectra. Sensitivities of 524 nm/RIU and 600 nm/RIU were achieved by transmission type sensors based on Au grating with *P* = 525 nm and Ag grating with *P* = 600 nm, respectively [33,34]. According to the above mentioned literatures, we can find that the sensor sensitivities are almost equal to the grating periods (S≈P), which is in good agreement with our theoretical calculations for sensors working at long wavelengths with |m|=1.

## 3. Experimental Demonstration

**Figure 5:**
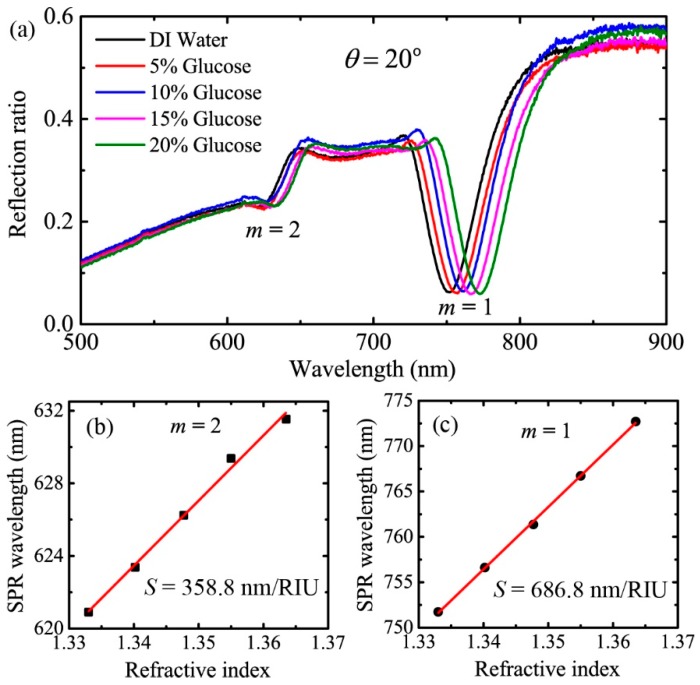
(**a**) The reflection spectra of the analytes experimentally measured at the incident angle of 20 degrees, (**b**,**c**) are the SPR wavelength as a function of the refractive index for diffraction orders of *m* = 2 and *m* = 1, respectively. The red lines are linear fits of the experimental data.

In order to further demonstrate our theoretical analysis, we experimentally measured the sensitivity of an Al grating-based sensor over a large wavelength range. The experimental setup is basically the same as that sketched in Figure 1. Detailed information of the setup can be found in our previous paper [35]. The grating was obtained by peeling off the metal layer from a SONY brand Digital Versatile Disc-Recordable (DVD-R) disc manufactured by Sony corporation in Taiwan, China. Here, the grating period and the modulation depth were 720 nm and 77 nm, respectively, according to the atomic force microscope image. Gratings fabricated by other two companies, namely Unisplendour corporation limited-company (UNIS) and the ANV series of Riteck corporation (ANV), were also measured. The grating periods for UNIS brand DVD-R disc made in Jiangsu, China and ANV brand DVD-R disc made in Taiwan, China were 724 nm and 727 nm. The modulation depths were 86 nm and 90 nm. As can be seen, the grating parameters of all the discs were similar. Gratings obtained from DVD-R discs of different manufacturers were proved to excite high quality SPR, and they were suitable to be applied in SPR sensors. In addition, the metal gratings fabricated by our method is stable for a long period of time. SPR response was measured for gratings stored in ambient conditions for more than 2 months. Deionized (DI) water and glucose solutions with concentrations of 5%, 10%, 15%, and 20% were used as the analytes. For instance, the reflection spectra measured at the incident angle of 20° are shown in Figure 5a. As can be seen, there are two absorption dips in the spectra. The deep resonance at the wavelength around 760 nm was caused by the excitation of SPR for the first diffraction order, and the shallow one around 625 nm was caused by SPR excitation for the second diffraction order. Extracting the SPR wavelengths from the reflection spectra, we can plot the SPR wavelength as a function of the refractive index of the analyte. The results are shown in Figure 5b,c, where the wavelength is linearly dependent on the refractive index. The sensitivities are then obtained by linearly fitting the experimental data. Therefore, for θ=20°, the experimental sensitivities are 686.8 nm/RIU and 358.8 nm/RIU for *m* = 1 and *m* = 2, respectively.

**Figure 6:**
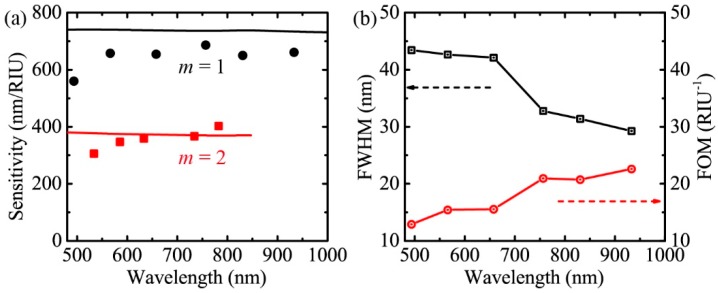
(**a**) Comparison of the experimental sensitivity and the theoretical sensitivity under the condition of *P* = 720 nm, $n_{a}=1.3402$. The black circles and red squares are experimental data. The black and red lines are theoretical data. (**b**) The *FWHM* and *FOM* as a function of the resonant wavelength.

Following the above procedure, we obtained the experimental sensitivities for 5% glucose solution at incident angles of 6°, 14°, 20°, 30°, 40°, and 50°. The results are shown as the black circles and red squares in Figure 6a for the first and second diffraction order, respectively. The theoretical sensitivities under the condition of *P* = 720 nm, $n_{a}=1.3402$ are also plotted in Figure 6a as a comparison. For instance, the theoretical value of the sensitivity for m=1 is 737 nm/RIU and that for m=2 is 372 nm/RIU at the resonant wavelength of 700 nm. We can see that the experimental sensitivity does not vary considerably across the analyzing wavelength range for each diffraction order, and the sensitivity for *m* = 1 is much higher than that for *m* = 2. These characteristics are consistent with the theory. The experimental data are slightly lower than the theory, which may be due to the imperfect alignment of the optical elements and experimental uncertainties. To further confirm the theoretical calculations, it is better to compare sensitivities of sensors with different grating periods. We are working on this and trying to experimentally fabricate metal gratings with various periods.

Other than the sensitivity, the figure of merit (*FOM*) is another important parameter to characterize sensor performance, which is defined as *FOM = S/FWHM* (the full width at half maximum of the absorption dip). The experimentally measured *FWHM* for 5% glucose solution ($n_{a}=1.3402$) with *m* = 1 and the corresponding *FOM* are shown in Figure 6b. The *FWHM* of the resonance dip decreases with increasing wavelength, which results in increasing *FOM*.

The sensitivity is calculated according to the wave vector matching. Since the grating wave vector is only dependent on the period and is independent of the modulation depth, we expect that the modulation depth will not influence the sensitivity, while the modulation depth will certainly influence the absorption line width and absorption depth of the reflection spectra. Therefore, the *FOM* of the sensor will be influenced. These influences cannot be solved by our analytical expression and can be solved by some numerical models.

## 4. Conclusions

In conclusion, we have derived analytical expressions for the sensitivity of SPR sensors based on metal gratings with wavelength interrogation. According to the theoretical analysis, the sensitivity increases with increasing wavelength and is saturated beyond a certain wavelength for Au and Ag gratings, while it is almost constant for Al gratings in the wavelength range of 500 to 1000 nm. More importantly, the grating period and the diffraction order dominate the value of sensitivity. Higher sensitivity is possible for SPR sensors with a larger grating period and lower diffraction order. At long wavelengths, a simple expression of *P*/|*m*| can be used to estimate the sensor sensitivity. Moreover, we experimentally measured the sensitivity of a SPR sensor based on an Al grating and compared the results with the theory. The wavelength and diffraction order dependence of sensitivity obtained in the experiments are consistent with the theory. The theoretical analysis presented in this paper will guide the optimization of system parameters for SPR sensors. For example, according to the theoretical analysis, a SPR sensor using a metal grating with a period of 1200 nm can achieve a previously unreported high sensitivity of 1200 nm/RIU.
